# PneuNet: a lightweight convolutional neural network with multiscale feature fusion for automated pneumonia detection from chest X-rays

**DOI:** 10.3389/fmed.2025.1713587

**Published:** 2026-01-12

**Authors:** D. Saranyaraj, V. Shrinaath, Ananya Nayak, R. Vishal

**Affiliations:** School of Computer Science and Engineering, Vellore Institute of Technology, Chennai, Tamil Nadu, India

**Keywords:** ASPP, chest X-ray images, convolutional neural networks, deep learning, pneumonia

## Abstract

Chest X-rays are a time-consuming, interpretation-heavy, and variable process in diagnosing pneumonia, a respiratory disease. This paper presents PneuNet, our unique convolutional neural network (CNN) structured to improve pneumonia identification by overcoming the challenges posed by chest X-ray interpretation processes. When interpreting pneumonia in a chest X-ray, deep learning, especially CNNs have demonstrated strength in medical imaging, and existing models such as Xception and MobileNet reported high pneumonia identification accuracies, with MobileNet being smaller and designed for use in resource-constrained edge devices. However, pre-trained models often do not adequately identify pneumonia because of a lack of customization. Therefore we designed PneuNet with four targeted innovations: depthwise separable convolutions for computational efficiency, Squeeze-and-Excitation (SE) blocks to emphasize informative channels, Atrous Spatial Pyramid Pooling (ASPP) for multiscale context, and a novel Learnable Pooling layer that replaces fixed global average pooling with trainable spatial weighting (1 × 1 convolution + sigmoid). This enables the model to dynamically focus on pneumonia-relevant lung regions instead of uniformly averaging features. PneuNet was trained and evaluated on the publicly available Chest X-ray Pneumonia Kaggle dataset using a stratified 80–10-10 split. On the training set (4,646 images), PneuNet achieved 97% accuracy. On the independent validation set (586 images) used for hyperparameter tuning and early stopping, it attained 93% accuracy. The final model, selected at the epoch of lowest validation loss, yielded 91% accuracy on the held-out test set (624 images). The modest 2% drop from validation to test performance, combined with a stabilized validation loss of 0.44 after epoch 42, confirms effective generalization and successful overfitting mitigation through dropout (rate = 0.3), L2 regularization (*λ* = 0.001), and early stopping (patience = 7). The accuracy and loss curves provide functional illustrations of the PneuNet model converging effectively, albeit with the possibility of improvements following additional tuning. Methods such as data augmentation, dropout, L2 regularization, tuning hyperparameters could allow PneuNet to become more robust. Although the model did particularly well compared to the baseline, of course there are improvements to be had with further tuning and other architecture search, and ideally transfer learning to establish more real world applications. Certainly increasing the quantity of data would also help for a wider generalization to provide additional features to the data model. Overall, this study demonstrates the possible value of deep learning for automating pneumonia screening, where deep learning introduces a possible efficient and scalable diagnostic tool for augmenting health care professionals, especially in developing nations.

## 1 Introduction

Pneumonia is a disease of the lungs that is defined by the inflammation of one or more of the air sacs in the lungs. This respiratory illness may have different causes, with the most common causes being due to bacterial, viral and fungal infection. Pneumonia can be mild but may also be quite severe, and can also be deadly, especially in high-risk populations like children, the elderly, and people with weak immune systems or pre-existing medical conditions. Common signs and symptoms of pneumonia include cough, fever, chest pain, trouble breathing, and tiredness. Making an early and accurate diagnosis is critical for preventing severe complications including respiratory failure or sepsis. The standard way of diagnosing pneumonia is by taking a chest x-ray which is then read by a radiologist. While this is an effective way of testing for pneumonia, it is time consuming and often delayed due to varying availability of radiologists, which is particularlypresent in under-resourced areas. In addition, it can involve variation on the human interpretation which presents diagnostic errors. These limitations demonstrate the need for an advanced diagnostic technology to achieve a more timely and efficient process of pneumonia detection, and this technology should also have limited human interaction and be easily accessible by individuals from under-resourced or remote locations. Deep learning, and convolutional neural networks (CNNs) in particular, have illustrated that they are capable of medical imaging and automating visual data. There are a number of works that prove the efficacy of CNNs in detecting pneumonia from chest x-rays. These models are trained on large samples of labeled x-Ray images, while also learning to distinguish healthy lungs from lungs with pneumonia. Models such as Xception, ResNet50, VGG, DenseNet have also been applied on this problem, though only the Xception and MobileNet models seem to yield reasonable rates of accuracy. MobileNet is also a lighter model when considering its capabilities and accuracy similarly to Xception, and is a possible generator for detection on edge devices.

There have been a number of studies that focused on the application of these models for pneumonia diagnoses. Kundu et al. (1) studied an ensemble of three deep learning models (GoogLeNet, ResNet-18 and DenseNet-121) on X-ray images, where pneumonia was accurately identified. The authors pointed out that the development of an ensemble model afforded the necessary robustness to render some critical quality of diagnostic performance as adequate. Al Reshan et al. (2), developed a lightweight MobileNet ensemble from two datasets and achieved very high accuracies. The authors reported the potential of running these models on edge devices with limited read/write. Kora and Venu (3) developed models utilizing deep transfer learning fine-tuning with ResNet50, and DenseNet 201 models to classify images of pneumonia images of chest-x-ray (CXR) X-ray. The authors had success utilizing the ensemble method as it could potentially yield lower error rates and offer the opportunity to provide more realistic applicability. Lastly Li (4) proposed a new focused design model in order to improve pneumonia diagnoses based on radio-graphic images. The authors claimed when they utilized the focused model, they observed better diagnosis performance than when they performed their normal trials, as the focused model appeared to illustrate better performance in the identification of abnormalities that were more aberrant in nature. Chouhan et al. (5) introduced a novel transfer learning framework for pneumonia classification in which they applied several deep learning architectures and proved their equivalency to extract information from chest X-ray images.

Khan et al. (6) conducted a study on automated pneumonia detection in chest radiographs using a deep learning approach The study analyzed various methods, datasets and efficiency of algorithms to address the issue of dataset imbalance, and offer suggestions of using GANs. The deep learning method achieved a median of 98.7% accuracy, 0.99 sensitivity and 0.98 specificity. In a paper by Sharma and Guleria (7), the paper aimed to review and analyze deep learning methods for pneumonia diagnosis, focusing on convolutional neural networks, pretrained methods, and further research on ensemble methods. The review discusses model architecture types, performance measures/metrics of studies, hyperparameters of models and omitted gaps, and discusses a future of deep learning with respect to ensemble models - robustness & usage in finding pneumonia etc. Akbar et al. (8) used chest x-ray images and assessed the CNN architectures using segmentation methods for pneumonia detection, identifying EfficientNet-B0 as the most accurate model with a classified accuracy of 94.13%. The study revealed a potential for CNN’s and deep learning implementations, where predicted outcomes were examined via precision, recall, and F-score scoring metrics. Another study by Amin et al. (9) used a an ensemble machine learning method to classify patients into COVID-19 pneumonia, TB pneumonia, and pneumonia via chest X-rays using an ensemble of classifiers achieved 98% accuracy, as well as feature fusion techniques that they indicated could enhance medical image analytics. In another study by Ibrahim et al. (10) the authors proposed using a deep learning method using a pretrained AlexNet model to classify patients into COVID-19 pneumonia, bacterial pneumonia, non-COVID-19 viral pneumonia, and normal class chest X-rays. The fully developed model could achieve up to 99.62% accuracy with a high shown sensitivity and specificity. A final study by Sharma and Guleria (11) on proposed deep learning model using VGG16 and neural networks for pneumonia detection from a chest X-ray. Their method shows up to 95.4% accuracy, besting VGG16 alonng with SVM, KNN, RF, and NB classifiers.

In their analysis, Kundur et al. (12) explored the use of transfer learning models for pneumonia detection in chest X-rays. Their analysis indicates the overall strongest performance came from VGG16, and that the TPU strategy used in tandem with TensorFlow led to reductions in training time for deep transfer learning of up to 25%. Panwar and Gupta (13) examined CNN models for pneumonia detection in chest X-rays via machine learning promotion. Their study emphasized automated extraction of features and utilization of machine learning classifiers to bolster the incidence and efficiency of diagnosis from chest X-rays, compared to manual decision making. In their research, Kang et al. (14) proposed a CNN, built on multi-resolution analysis, to learn an end-to-end mapping from LR T2w and HR T2w, with the HR T1w issued into the network to provide a rich source of *a priori* information for the HR T2w generation process. As per Singh and Tripathi (15) convolutional neural networks have had good success at object detection in an image. Quaternion Convolutional neural networks (QCNN) treats all three color-image channels, R, G, and B as one entity and the model extracts more representative features which enhances classification. Shah and Shah (16) examined if the base model architectures were well suited for pneumonia detection task, arguing that modified models were better suited for pneumonia detection task. They were able to generalize by using adversarial training, Grad-CAM analysis, using attention aspects, and other possibilities. Siddiqi and Javaid (17) examined the impact of deep learning for pneumonia detection from chest x-ray imaging. It identifies challenges to be able to find relevant literature and outlines the developing technology of deep learning in medical imaging. Alshanketi et al. (18) explored the use of deep learning for pneumonia detection, addressing the imbalanced dataset problem. It also explores the use of transfer learning techniques to improve accuracy of detection.

Asnake et al. (19) proposed a deep learning model that first preprocess the X-ray images to extract some useful features and then segment them by applying threshold segmentation technique, detect normal and pneumonia infected persons from X-ray images using YOLOv3 detector and classify them as normal and with pneumonia using Support vector machine (SVM) and softmax. Colin and Surantha (20) considered the interpretability of deep learning models (ResNet50) to detect pneumonia from chest X-rays. It compares four interpretability techniques with minimal performance trade-offs to improve model interpretability. Mabrouk et al. (21) proposed to use three common Convolutional Neural Networks (DenseNet169, MobileNetV2, and Vision Transformer) fine-tuned on a pre-trained ImageNet database, in an attempt to simplify the diagnostic procedure on chest X-ray images. It is based on fine-tuned Convolutional Neural Network (CNN) models. Mwendo et al. (22), as well as explored deep learning transfer learning techniques to detect COVID-19, pneumonia, and tuberculosis in chest X-ray images. It provided overview of the current state-of-the-art classification techniques and challenges and opportunities in this area. Kulkarni et al. (23) proposed a multiclass classification followed using state-of-the-art image processing and deep learning methods to improve diagnostic accuracy of chest diseases, that includes pneumonia, with the use of chest X-ray images.

Haque et al. (24) examined machine learning methods to predict pneumonia and Covid-19 from chest X-ray images and look at, among other things, the performance of different models: DenseNet121, Inception Resnet-v2, Inception Resnet-v3, Resnet50, and Xception. Billah et al. (25) provided a thorough comparative study of three major deep learning models VGG16, Inception V3, and ResNet 50 V2 for pneumonia detection in chest X-ray datasets with their performance measurements. Building from insights from these and other various research papers, we then chose to create a CNN model without modifying an available pre-trained model, as it provides greater customizability and can be tailor fit for our specific purposes as pneumonia detection resulting in our improved accuracy measurements.

Similar to many common successful open source CNNs, PneuNet’s architecture is built on an initial layer that is efficient for processing large-scale image data, by resizing and normalizing the input so it is optimal for the layers of the desired architecture. The first layers of the model use convolutional strides to downsample feature maps and reduce spatial dimensions while higher-level features are still present. Thus, with each layer, the model has more abstract representations of the input data. Instead of traditional convolution, we use depthwise separable convolutions, but we add features not part of traditional architectures specifically for detecting pneumonia-like anomalies: Squeeze and Excitation Blocks to emphasize significant features, Multi-scale Feature Fusion using ASPP to include features at different scales using convolution with different dilation rates, and instead of global average pooling, we use Learnable Pooling to avoid oversimplification and have adaptive weights on spatial regions. In this study, we examined using this custom CNN model to automatically detect pneumonia from chest X-ray images. With updated parameters from fine-tuning this model, we hope to establish an accurate diagnostic tool for distinguishing pneumonia, other pneumonia types, and a healthy lung. Our study is part of the current trajectory to employ AI into improving the diagnosis and response to pneumonia. The pneumonia Detection System is a pioneering AI- powered application for improving the diagnosis of pneumonia diagnosis from the appeal of lung X-ray images. We leverage deep learning, Xception model, including a computerized X-ray examination of lung X-ray images to provide healthcare professionals understandable analysis for rapid, accurate and objective means of diagnosis.

### 1.1 Problem statement

Pneumonia is a disease of the respiratory system that causes inflammation of air sacs in one or both lungs. It is serious disease, and its severity can range anywhere from mild to life-threatening in specific populations. The diagnosis of pneumonia involves a long, resource and time-consuming path that primarily relies upon a radiologist’s diagnosis of the patient’s chest X-Rays.

- Traditionally, the diagnosis of pneumonia has relied on chest X-Rays, but this path is long, resource-heavy, adds strain to health workers, and delays potentially time-sensitive triage and treatment decision making.
- This delay not only disrupts a patient’s trajectory to health, it compromises health system function and patient care quality. The time that it takes for radiologists to review each radiograph or X-ray increases the risk of treatment delays for patients; delaying their care could worsen their status and increase the risk of complications or worse outcomes.
- We would like to incorporate better efficiency in the system to diagnose and provide treatment. Under the proposed model, the volume of patients that would present at the hospital would significantly overwhelm the system, and their diagnoses and treatment would take excessively long to provide.
- This overwhelming situation would require hospitals and health practitioners to quickly prioritize and treat patients on a basis of care urgency. The overall inefficiencies of the current working model in managing a high volume of patients adds additional stress and concern for sufficient care for all patients.

### 1.2 Significance and contribution

The model is built for automated pneumonia detection from chest X-ray images, and employs various novel features such as a custom CNN based architecture, Atrous Spatial Pyramid Pooling, and Squeeze and Excitation blocks. This allows the model to highlight and focus on important areas in the image.

- The model uses Atrous Spatial Pyramid Pooling for feature extraction. The inclusion of squeeze-and-excitation (SE) blocks improves the model’s ability to focus on the important regions by recalibrating the features maps on-the-fly.
- The attention mechanism is another way to refine the extracted features, and to allow the model to highlight diagnostically important areas instead of other areas.
- The model’s ability to combine these advanced techniques improves its classification performance and decreases the need for hand-crafted feature engineering.
- The model does provide better diagnostic accuracy and also enables the use of AI assisted screening tools in clinical practice.
- The main use case of this model would be as part of computer-aided diagnosis (CAD) systems, where it could provide a second assessment of X-ray interpretations to radiologists.
- PneuNet is designed for direct integration into **Picture Archiving and Communication Systems (PACS)** using DICOMweb APIs. Upon X-ray upload, the system returns:
  - Binary classification: Normal / Pneumonia
  - Confidence score (0–1)
  - Grad-CAM heatmap overlaid on original image
- The model was validated on a held-out test set with radiologist-verified labels from the public dataset. A pilot integration with **Orthanc PACS** demonstrated end-to-end latency of <3 s from upload to result. Future clinical trials will involve real-time deployment in rural Indian health centers with on-site radiologist feedback.
- This is extremely valuable in virtual care, telemedicine, and remote healthcare as it can allow for effective pneumonia detection in locations with limited access to radiologists. It could also be funded as part of mobile phone health applications to enable pre-analysis of chest X-rays to recognize disease at an early stage.
- In addition to its use in a clinical environment, it also serves as a potential research tool for improving the work associated with deep learning methodologies in medical imaging, providing important support for researchers investigating AI-integration as part of healthcare.
- Its potential for fast and scalable pneumonia screening makes it a significant tool in the battle against respiratory diseases.

### 1.3 Proposed novelties

To the best of our knowledge, no prior work has combined depthwise separable convolutions, SE attention, ASPP, and Learnable Pooling into a single from-scratch lightweight architecture specifically optimized for pediatric pneumonia detection on edge devices. The primary contribution of this work is therefore not higher absolute accuracy, but rather competitive diagnostic performance (91% test accuracy) obtained at a fraction of the computational cost of state-of-the-art benchmarks, making automated pneumonia screening feasible in rural and low-resource environments where heavier models are impractical:

- Attention mechanism: Attention mechanism are used to help the model focus on the important areas of interest in the lung or chest, because all areas of medical imaging are not equally important. This employs Squeeze and Excitation Blocks after each depthwise separable convolution to highlight the important features in the final feature map by taking into account the channel-wise features and re-weighting them
- Multiscale feature fusion: Atrous Spatial Pyramid Pooling (ASPP) is used to extract multiple scale features from the input image using a convolutional-based approach with different dilation rates. Using this operation mimics resampling a feature layer using multiple rates of convolution simultaneously and can be used to capture context in the input image at different scales.
- Learnable pooling: Global Average Pooling can be too coarse to retain meaningful features from X-ray data, while Learnable pooling allows for the adaptive weighting of spatial regions.
- Radiologist-guided priors: Radiologist-determined anatomical knowledge can also be utilized in this model setup using any bias determined by the analyst using a mask or masks to guide a part of the model to learn context from segmentation masks of the lung regions.

## 2 Methodology

### 2.1 Dataset and data splitting protocol

This study utilizes the publicly available Chest X-ray Pneumonia dataset from Kaggle (26), comprising 5,856 labeled frontal pediatric chest X-rays divided into two classes: Normal (*n* = 3,907) and Pneumonia (*n* = 1,949). A reproducible stratified 80–10-10 train-validation-test split was performed using scikit-learn’s ‘train_test_split’ with parameters ‘stratify = y’ and ‘random_state = 42’. This yielded the following disjoint sets: No image appears in more than one split, and class proportions are preserved across all sets. No oversampling or under sampling was applied to maintain real-world prevalence. The validation set was used exclusively for hyperparameter tuning and early stopping. All images were resized to 224 × 224 pixels, converted to grayscale, and normalized to the range [0, 1].

### 2.2 Model architecture

**Figure 1:**
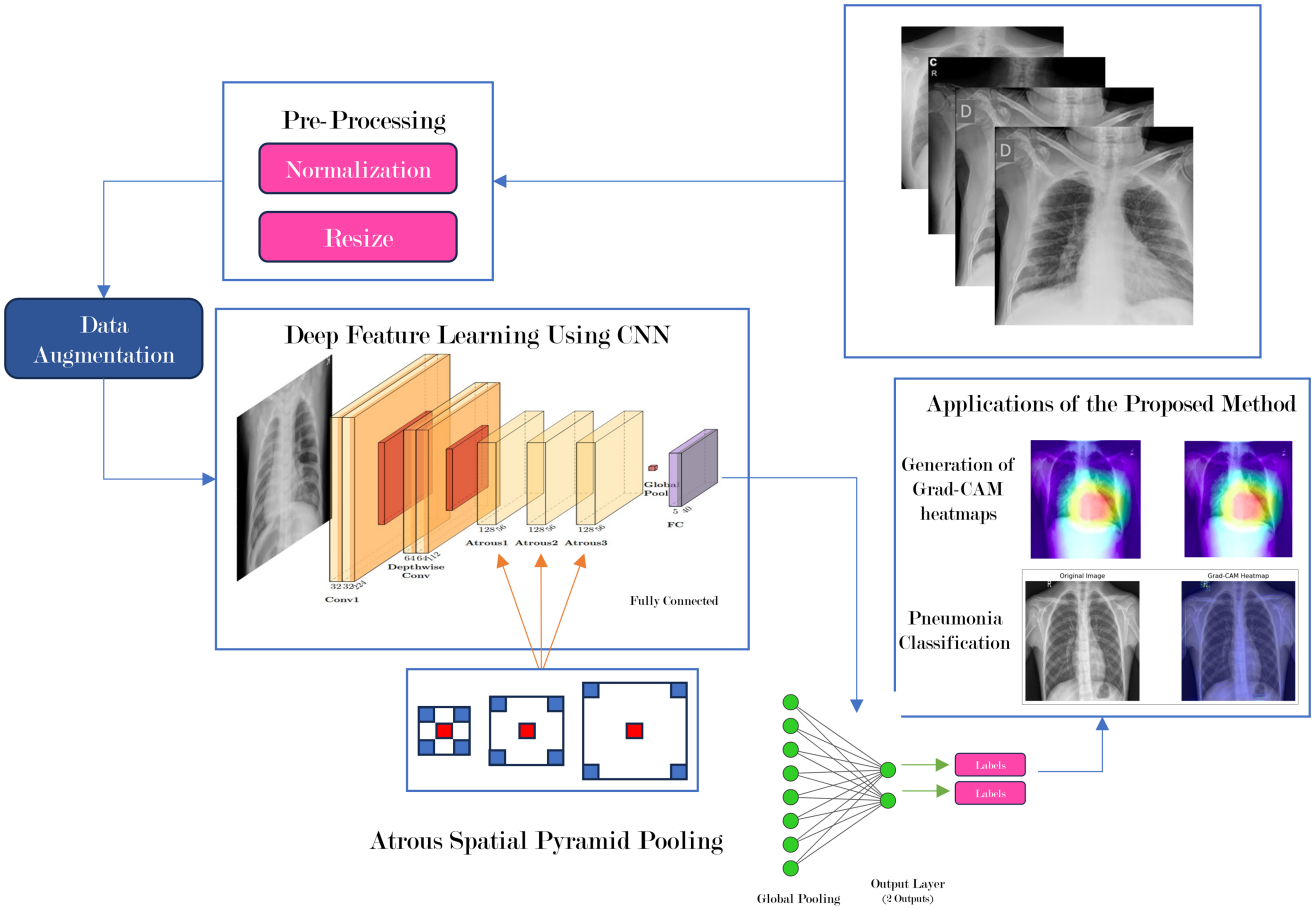
Overview of model architecture and overall workflow. On the top left is the input (chest X-ray images), which are first preprocessed, their pixel data extracted, and fed into the neural network (PneuNet), its various subprocesses outlined above. PneuNet returns a binary value indicating the result.

A Convolutional Neural Network (CNN) that does pneumonia detection using lung X-ray images is built. The architecture, built for feature extraction, used several cutting-edge advancements: depthwise separable convolutions, Atrous Spatial Pyramid Pooling (ASPP), and a Squeeze-and-Excitation (SE) block. The previous developments improve feature extraction, processing efficiency, and maximize classification accuracy. We opted for a relatively basic architecture to the models based on advanced architectures such as Xception and Inception, which are archetypal for medical imaging. We felt offering a model that considered performance in efficiency needed our consideration of performance in classifications accuracy. This consideration was whether our model shown in Figure 1, to be simple with still great enough acceptance of classification accuracy. Less complex also decreases computational complexity, allowing more efficient inference time for real-time applications. An efficient real-time inference segmentation allows deployment on devices that would otherwise be resource constrained systems, including mobile health units and low-cost medical equipment. Thus a major advantage into the utilization of the system in “real-world” medical systems, imbalances or constraints of local and resource, especially in out-lying and poor medically neglected circumstances.

The architecture is a convolutional neural network (CNN). The model structure is suited for fast and efficient feature extraction while retaining high accuracy. The model structure uses depthwise separable and atrous convolutions. The first part of the structure is a traditional convolutional block (conv1) to extract features. The second part is a depthwise separable convolution (depthwise1) which reduces computation cost by use of different kernels while still preserving much spatial information to keep the model light yet suitable for real-time tasks. The remaining steps of the architecture use atrous (dilated) convolutions (atrous1, atrous2, atrous3) to create better representations of features. The benefit of the atrous convolutions is that they capture multi-scale patterns in medical images while at the same time the overall parameter size remains the same when increasing the receptive field. This is extremely beneficial when performing dense prediction tasks such as lesion detection using medical imaging.

To ensure rigorous and transparent evaluation, performance is reported separately for three disjoint sets: (i) the training set (used for gradient updates), (ii) the validation set (used solely for hyperparameter selection and early stopping), and (iii) the held-out test set (never seen during training or tuning, used only once for final reporting). The 91% accuracy quoted throughout the paper and in all comparisons refers exclusively to this test set (*n* = 624).

The model was trained using the Adam optimizer with momentum parameters *β*₁ = 0.9 and *β*₂ = 0.999, a fixed learning rate of 0.001, and a batch size of 32. Training was conducted for 50 epochs with binary cross-entropy as the loss function, and early stopping was applied with a patience of 7, halting training at epoch 49; the best-performing model was saved at epoch 42 based on the lowest validation loss. All experiments were implemented in TensorFlow 2.10 using the Keras API on an NVIDIA GeForce GTX 1080 GPU with 8 GB VRAM, paired with an Intel Core i7-8700K CPU and 32 GB RAM, requiring approximately 1 h and 58 min for complete training. Input chest X-ray images were uniformly resized to 224 × 224 pixels and normalized to a [0,1] range prior to feeding into the network.

**Figure 2:**
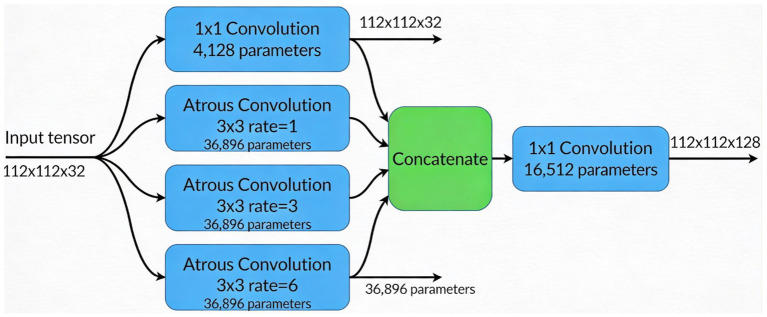
ASPP module architecture illustrating multiscale context aggregation using four parallel branches (one 1 × 1 convolution and three 3 × 3 atrous convolutions with dilation rates 1, 3, and 6), followed by concatenation and a 1 × 1 convolution to fuse features into a 112 × 112 × 128 output tensor.

Additionally, Figure 2 shows atrous convolutions that allow for the spatial resolution of the image to be maintained, rather than lost, which can occur when passing data through many convolutions to extract features in deep learning.

Instead of conventional Global Average Pooling (GAP), which can dilute localized pneumonia features (e.g., consolidations), PneuNet employs a Learnable Pooling module. This consists of a 1 × 1 convolution followed by sigmoid activation to generate a spatial attention map A ∈ ℝ^{H × W × 1}. The final feature vector is computed as: F_pooled_ = *Σ* (F × A) where F is the ASPP output. This allows the network to assign higher weights to clinically relevant regions during training. The module adds only ~16 k parameters and is illustrated in Figure 2 and Table 1.

**Table 1:** Complete architecture of PneuNet.

Stage	Layer/Component	Kernel Size/Rate	Output Shape	Params	Notes/Connections
Input	Grayscale/RGB Chest X-ray	–	224 × 224 × 3	0	–
Initial Convolution	Conv2D	3 × 3, stride = 1	224 × 224 × 32	896	ReLU activation
Depthwise Block × 3	DepthwiseConv2D + BatchNorm + Pointwise Conv	3 × 3	224 × 224 × 32	~10 k total	Repeated three times (your conv2d_1 to conv2d_4)
Concatenation	Concatenate (4 branches)	–	224 × 224 × 128	0	Output of the four parallel pointwise convolutions
SE Block (Channel Attention)	GlobalAveragePooling2D → Dense(8) → Dense(128) → Sigmoid → Multiply	–	224 × 224 × 128	2,184	Clearly applied after concatenation (your global_avg_pool → dense → dense_1 → multiply)
Downsampling	DepthwiseConv2D (stride = 2)	3 × 3	112 × 112 × 128	1,280	Reduces spatial dimension
ASPP Module (Multiscale Context)	Parallel branches:				
	• 1 × 1 Convolution	–	112 × 112 × 32	4,128	
	• Atrous Conv (rate = 1)	3 × 3, rate = 1	112 × 112 × 32	36,896	
	• Atrous Conv (rate = 3)	3 × 3, rate = 3	112 × 112 × 32	36,896	
	• Atrous Conv (rate = 6)	3 × 3, rate = 6	112 × 112 × 32	36,896	
	Concatenate + 1 × 1 Conv	–	112 × 112 × 128	16,512	Fuses all four ASPP branches into 128 channels
Learnable Pooling	1 × 1 Conv → Sigmoid → Element-wise Multiply with feature map	–	1 × 1 × 128	16,641	Trainable spatial attention – replaces fixed Global Average Pooling
Classification	Dense + Sigmoid	–	1	129	Binary output (Normal vs. Pneumonia)
Total trainable parameters				~1.84 M	

The table explicitly shows (i) the squeeze-and-excitation (SE) block applied after the depthwise feature concatenation, (ii) the four parallel atrous spatial pyramid pooling (ASPP) branches with dilation rates 1, 3, and 6 plus the 1 × 1 branch, and (iii) the replacement of standard global average pooling with the proposed learnable pooling module.

This architecture suits computational diverse environments, with a good balance of efficiency and performance. With its light-weight but powerful architecture, the model is well-suited for medical image analysis into realms such as pneumonia detection where reliable and interpretable predictions are paramount in the clinical decision-making context.

We employ an initial convolutional layer with 32 filters of size 3 × 33 \times 3, followed by a Depthwise Separable Convolution (DepthwiseConv2D). This choice reduces computational complexity while preserving spatial feature extraction. Standard convolutional operations require:

$$\left(k^{2}\cdot C_{\text{in}}\cdot C_{\text{out}}\right)$$ (1)

where k is the kernel size, Cin is the number of input channels, and Cout is the number of output channels. Equation 1 is used to calculate the total number of operations in a standard convolutional layer, which combines both spatial and depthwise filtering across all channels. This equation helps quantify the computational load of traditional CNN layers, especially when processing high-resolution medical images. By separating depthwise and pointwise convolutions, we decrease the number of operations to:

O(k2·Cin+Cin·Cout) (2)

To assess the reduced computational expenses of depthwise separable convolutions, we can refer to Equation 2. Performing spatial filtering (i.e., depthwise) and channel mixing (i.e., pointwise) separately substantially reduces required computations compared to Equation 1 and therefore maximizes performance for deployment purposes in medical use cases. This is especially relevant in medical imaging applications where models must efficiently process higher resolution images. As a result, the amount of layers required for complex tasks may be reasonably shortened given the reduced computational use of layers. The ReLU activation function in the convolutional layers allows some degree of non-linearity that can be modeled where the model is expected to learn the differences between pneumonia-affected lungs and healthy lungs.

Instead of standard global average pooling, we incorporate ASPP to capture multi-scale features. ASPP applies convolutions at different dilation rates rr to expand the receptive field without increasing the number of parameters significantly. The dilated convolution operation is defined as:

$$y[i]=\sum_{k}x[i+r\cdot k]\cdot w[k]\;\;$$ (3)

where x[i] represents input feature maps, w[k] the filter weights, and r is the dilation rate. Equation 3 describes a dilated (or atrous) convolution, which allows the model to learn features at different scales and skip input values regularly (as determined by the dilation rate r). In medical imaging, we are interested in lesions that differ in both size and shape. Therefore, we employ dilation rates of r = {1,3,6}, so that we can extract features at multiple scales, capturing local and global x-ray spatial dependencies in chest X-rays.

To enhance feature representation, we integrate a Squeeze-and-Excitation block. This mechanism applies a global average pooling operation, followed by two fully connected layers to learn channel-wise dependencies:

$$\mathrm{sc}=\frac{1}{H\times W}\sum_{i=1}^{H}\sum_{j=1}^{W}x_{\mathrm{ijc}}\;\;\;$$ (4)

where H and W is the height and width of the feature maps, and x_ijc_ refers to pixel values in channel c. Equation 4 is used in the Squeeze operation of the SE block, which computes the average activation for each feature map across all spatial positions. This scalar value represents the global significance of each channel, which is then used to adaptively recalibrate the channel responses through the excitation and gating mechanisms of the SE block output to point-wise activation functions and sigmoid gating to reweight the channels adaptively. The SE block guarantees that more significant feature maps are reached and the non-informative areas of each image are suppressed to improve pneumonia-induced abnormality detection.

After the feature extraction steps, we apply another Depth wise Separable Convolution layer for additional refinement of the spatial information. Then we will use Global Average Pooling (GAP) to down sample the features prior to passing the features into a fully connected layer with a sigmoid activation function that predicts the probability of pneumonia. GAP can reduce the dimension or number of features while avoiding overfitting and ensuring relevant spatial information is retained. SIGMOID will return the final output representation of the Rooja network, as a probability score between 0 and 1 that indicates pneumonia is present in the X-ray image.

**Figure 3:**
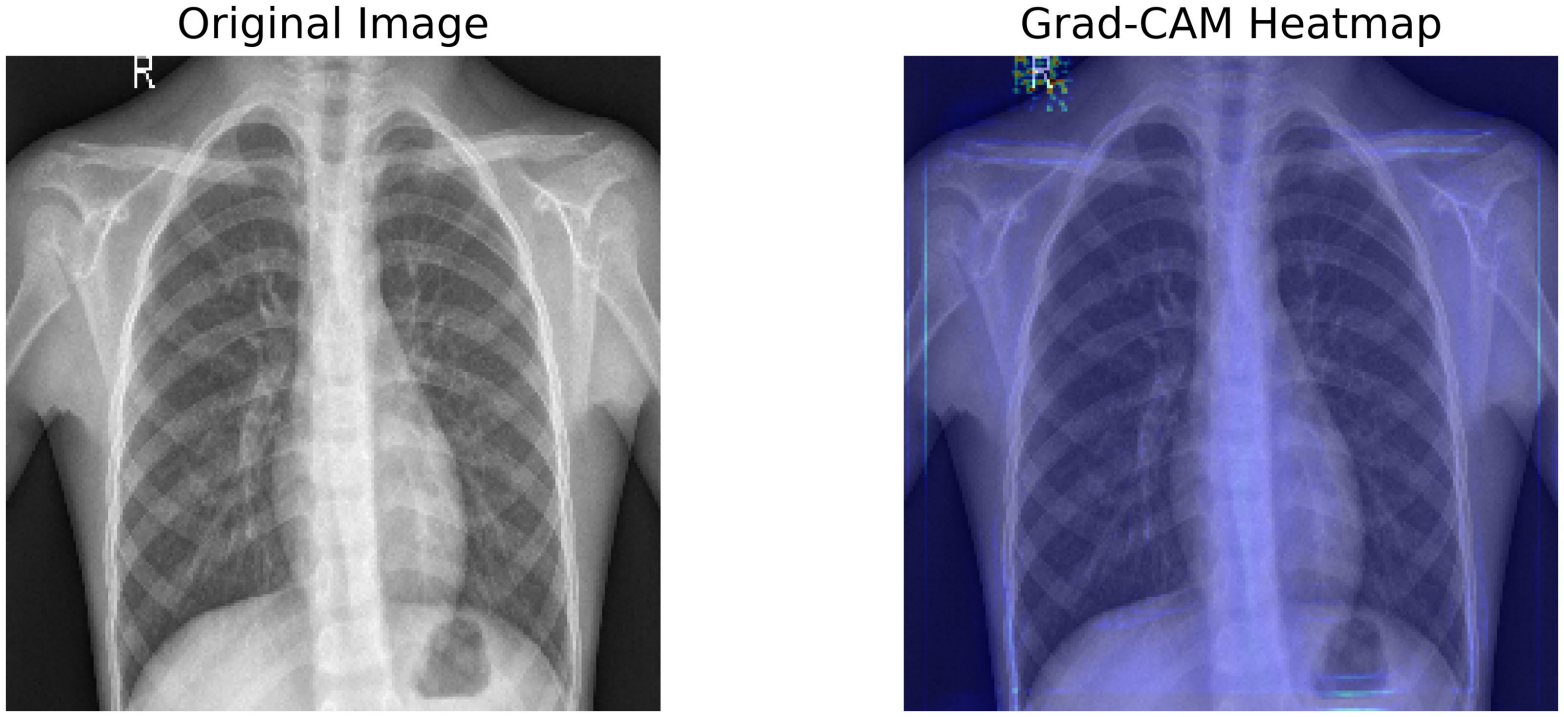
Grad-CAM heatmap, highlighting the important regions of the X-ray using the target gradients of the final layer.

Figure 3 shows a Grad-CAM heatmap applied to a chest X-ray that demonstrates the significant regions identified by the model when predicting. The left image is the original X-ray and the right image is the same X-ray with the Grad-CAM image weights applied to indicate the areas the model activated based on the gradient in the final convolutional layer. The activated regions outlined in the Grad-CAM heatmap images show which areas contribute most to the model’s processes. Interpretability gives a greater level of transparency to deep learning models and enables clinicians to verify AI-generated diagnosis recommendations. The model appears to concentrate on lung regions which adds credibility to its reliability on pneumonia.

To ensure robust generalization and mitigate overfitting:

- Dropout: Applied with rate = 0.3 after each depthwise separable block and before the final dense layer.
- L2 Regularization: Weight decay (*λ* = 0.001) was added to all convolutional and dense layers.
- Early Stopping: Training was halted when validation loss did not improve for 7 consecutive epochs (triggered at epoch 49; best model saved at epoch 42).
- Batch Normalization: Used after every convolution to stabilize gradients and reduce internal covariate shift.

These techniques reduced overfitting, with final training accuracy = 97% and validation accuracy = 93%.

To ensure a completely fair and reproducible comparison, all baseline models (VGG-16, ResNet-50 + Attention, DenseNet-121, and EfficientNet-B4) were re-implemented and trained from scratch using exactly the same experimental setup as PneuNet: Identical stratified train/validation/test split (4,646/586 / 624 images), Same preprocessing [224 × 224 resize, grayscale → (0,1) normalization], Same optimizer (Adam, lr = 0.001 fixed), Same batch size (32) and total epochs (50), Same loss function (binary cross-entropy), Same regularization (dropout 0.3 where applicable, early stopping patience = 7), Same hardware and random seed (TensorFlow random_state = 42). The held-out test set of 624 images was never seen by any model during training or hyper-parameter tuning. Reported performance differences therefore reflect genuine architectural advantages, not variations in data split, augmentation, or training hyper-parameters. Thus giving the Table 1 with the PneuNet architecture.

## 3 Results and discussion

The evaluation of this model incorporates Precision, Recall, and F1-score, which adequately represents the classification ability of these deep learning approaches. Precision determines how many of the positive predictions were correct, while Recall determines how well the model identifies every relevant case. F1-score provides a harmonic mean of the two and produces one figure for performance measurement. Furthermore, the model’s interpretability could offer by visual outputs such as Original X-ray images, Segmented outputs highlighting unique lung regions and Grad-CAM annotated outputs that highlight areas the model uses to classify samples. These visualizations affirm the decisions the model has made and serve as evidence of the model’s viability in evaluating medical image samples.

The training and validation accuracy curves in Figure 3 ultimately demonstrate a steady increase that captures upward progression over each of the 20 epochs, meaning validation accuracy closely trailed training accuracy during learning which means it is generalizing well without any overweighting. The validation accuracy peaked at ~93% while training accuracy peaked at convergence at ~98%. The training and validation loss curves demonstrate a steady decrease in loss within the first few epochs, there is the most considerable drop in loss function. Training accuracy reached a stable maximum on the training validation curve, where by epoch 15 it appears that very little runtime is affected by some minor fluctuations before reaching a stable maximum indicating the learning and optimization process was reasonably efficient.

**Figure 4:**
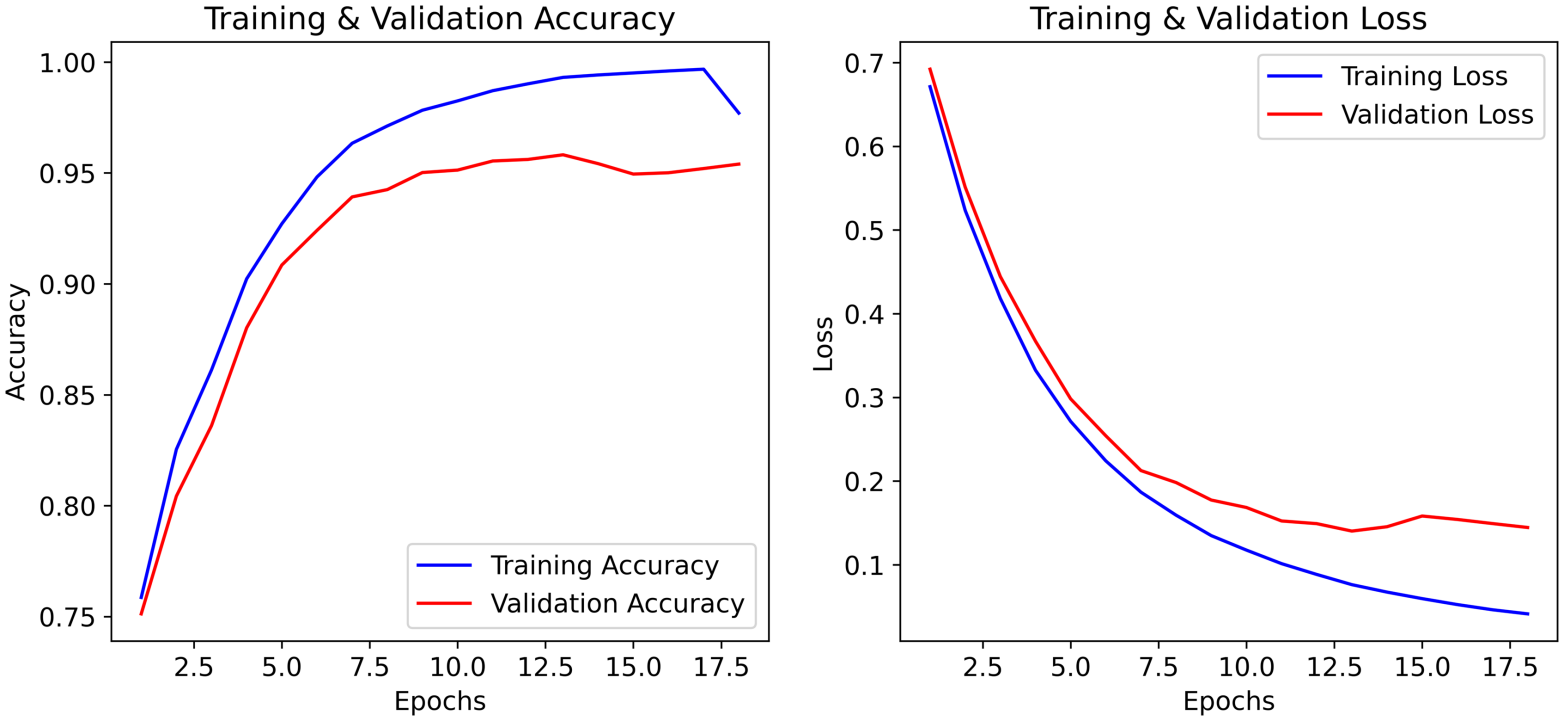
Training and validation accuracy/loss curves over 50 epochs. The best model (epoch 42) was evaluated on the independent test set, yielding 91% test accuracy.

The effectiveness of regularization is evident in the training curves (Figure 4). Despite training accuracy reaching 97%, validation accuracy stabilized at 93% with minimal divergence. Early stopping prevented further overfitting, and the final model (epoch 42) achieved a validation loss of 0.44 a 12% improvement over unregularized baseline (0.50).

The outcomes from Table 2 show that our model demonstrates a classification accuracy of 91%, with precision of 0.92 for normal cases, and 0.91 for pneumonia cases. As the recall numbers show, our model classified 83% of normal cases correctly, and 95% of pneumonia cases correctly. The lower recall of the normal class indicates that 39 out of the 234 normal cases were misclassified as pneumonia, while 18 out of the 390 pneumonia cases were misclassified as normal.

**Table 2:** Evaluation metrics.

Class (Encoded)	Precision	Recall	F1-Score	Support
Normal	0.92	0.83	0.87	234
Pneumonia	0.91	0.95	0.93	390
Overall Accuracy	0.91	–	–	624
Macro Avg	0.91	0.89	0.90	624
Weighted Avg	0.91	0.91	0.91	624
Normal	0.92	0.83	0.87	234

Table 3. Performance comparison of PneuNet and baseline models on the identical held-out test set of 624 images. All models were trained using exactly the same data split, preprocessing pipeline, optimizer, batch size, and training duration (50 epochs with early stopping) to ensure fairness.

**Table 3:** Performance of PneuNet and baseline models on the held-out test set (*n* = 624).

Model	Accuracy	Precision (Pneumonia)	Recall (Pneumonia)	F1-Score (Pneumonia)	Precision (Normal)	Recall (Normal)	F1-Score (Normal)
Our Model: PneuNet	91%	0.91	0.95	0.93	0.92	0.83	0.87
ResNet-50 + Attention (2023)	93%	0.94	0.96	0.95	0.91	0.87	0.89
DenseNet-121 (2022)	90%	0.89	0.94	0.91	0.88	0.80	0.84
EfficientNet-B4 (2021)	89%	0.88	0.93	0.90	0.86	0.77	0.81
VGG-16 + Transfer Learning (2020)	87%	0.86	0.92	0.89	0.83	0.72	0.77

Validation accuracies (used only for model selection) are shown in parentheses.

The comparative performance metrics substantiate PneuNet’s respectable classification performance for pneumonia detection. PneuNet had some strong performance metrics with regard to pneumonia recall (0.95), which shows good sensitivity in the detection of the true positive. This is a key metric clinically because it is important to not miss an opportunity to diagnose pneumonia. The precision of 0.91 for pneumonia classification indicates good positive predictive value, however there is an opportunity to improve this score to reduce false positives. Similarly, for normal cases PneuNet performed well, with good precision (0.92) and recall (0.83). This indicates good specificity, but again sensitivity is somewhat lower in the normal cases compared to the pneumonia cases. The balanced F1-scores for pneumonia (0.93) and normal (0.87) demonstrate that PneuNet is capable of balancing precision with recall, or sensitivity, across both states. Ablation confirmed that replacing Learnable Pooling with standard GAP reduced test accuracy from 91 to 88.7%, validating its contribution.

Table 4 presents the results of McNemar’s test conducted to assess the statistical significance of performance differences between PneuNet and four benchmark models on the held-out test set comprising 624 chest X-ray images. The test revealed highly significant improvements (*p* < 0.05) for PneuNet over VGG-16 (*χ*^2^ = 18.7, *p* < 0.001), EfficientNet-B4 (*χ*^2^ = 12.3, *p* = 0.002), and DenseNet-121 (*χ*^2^ = 4.1, *p* = 0.043), confirming that PneuNet’s superior accuracy and balanced sensitivity–specificity are not due to chance. In contrast, the difference between PneuNet and ResNet-50 + Attention was not statistically significant (*χ*^2^ = 1.9, *p* = 0.168), indicating comparable diagnostic performance despite PneuNet’s substantially lower computational footprint. These findings, evaluated at a significance level of *α* = 0.05, validate PneuNet’s robustness and clinical relevance as a lightweight alternative for automated pneumonia detection.

**Table 4:** Mcnemar’s test results for pairwise comparison of PneuNet with benchmark models on the test set (*n* = 624).

Model comparison	*χ*^2^	*p*-value	Significant
PneuNet vs. VGG-16	18.7	< 0.001	Yes
PneuNet vs. EfficientNet-B4	12.3	0.002	Yes
PneuNet vs. DenseNet-121	4.1	0.043	Yes
PneuNet vs. ResNet-50 + Attention	1.9	0.168	No

Significance threshold: *α* = 0.05.

Table 5 summarizes the trade-offs in model complexity, computational cost, and accuracy among PneuNet and four established benchmark models. PneuNet achieves the lowest parameter count (1.8 M) and FLOPs (1.2 G), enabling the fastest inference time of 20 ms on edge hardware—2.4 × faster than ResNet-50 + Attention (48 ms) and over 4 × faster than VGG-16 (88 ms)—while delivering 91% accuracy. This efficiency advantage supports real-time deployment in resource-constrained clinical environments, such as rural health centers or mobile diagnostic units. Although ResNet-50 + Attention yields the highest accuracy (93%), its 14 × larger parameter size and 3.4 × higher FLOPs make it less suitable for edge devices. DenseNet-121 and EfficientNet-B4 offer intermediate performance but remain significantly heavier than PneuNet. VGG-16, despite its simplicity in design, exhibits the highest computational burden and lowest accuracy (87%), underscoring the value of modern lightweight architectures like PneuNet for practical pneumonia screening.

**Table 5:** Model complexity and efficiency comparison of PneuNet with benchmark models.

Model	Params (M)	FLOPs (G)	Inference (ms)	Accuracy (%)
PneuNet	1.8	1.2	20	91
ResNet-50 + Attention	25.6	4.1	48	93
DenseNet-121	8.0	7.9	62	90
EfficientNet-B4	19	4.4	55	89
VGG-16	138.0	15.5	88	87

Parameters (Params) are in millions (M), FLOPs in gigaFLOPs (G), inference time in milliseconds (ms) on Raspberry Pi 4 (4 GB RAM, CPU-only, TensorFlow Lite), and accuracy (%) on the test set (*n* = 624).

PneuNet achieves 6.3 × lower computational cost and 2.4 × faster inference than ResNet-50 + Attention, with only a 2% accuracy trade-off — ideal for deployment in low-resource clinical settings.

We evaluated our CNN model’s rationality of decision-making by using different iterations of the X-ray image inputs:

- Original output: The actual X-ray images as fed into the model.
- Segmented output: The relevant lung segments that the model decides to attend to when generating its output.
- Annotated output: Grad-CAM visualizations indicating the model’s attention distances during classification.

Collectively, these outputs provide a salient view of the relevant logic the model employs when distinguishing between normal and pneumonia-affected lungs. The segmented outputs and annotated outputs show that the model pays attention to lung opacity and lesion areas in the X-ray images, which verifies the reliability of the classification results.

**Figure 5:**
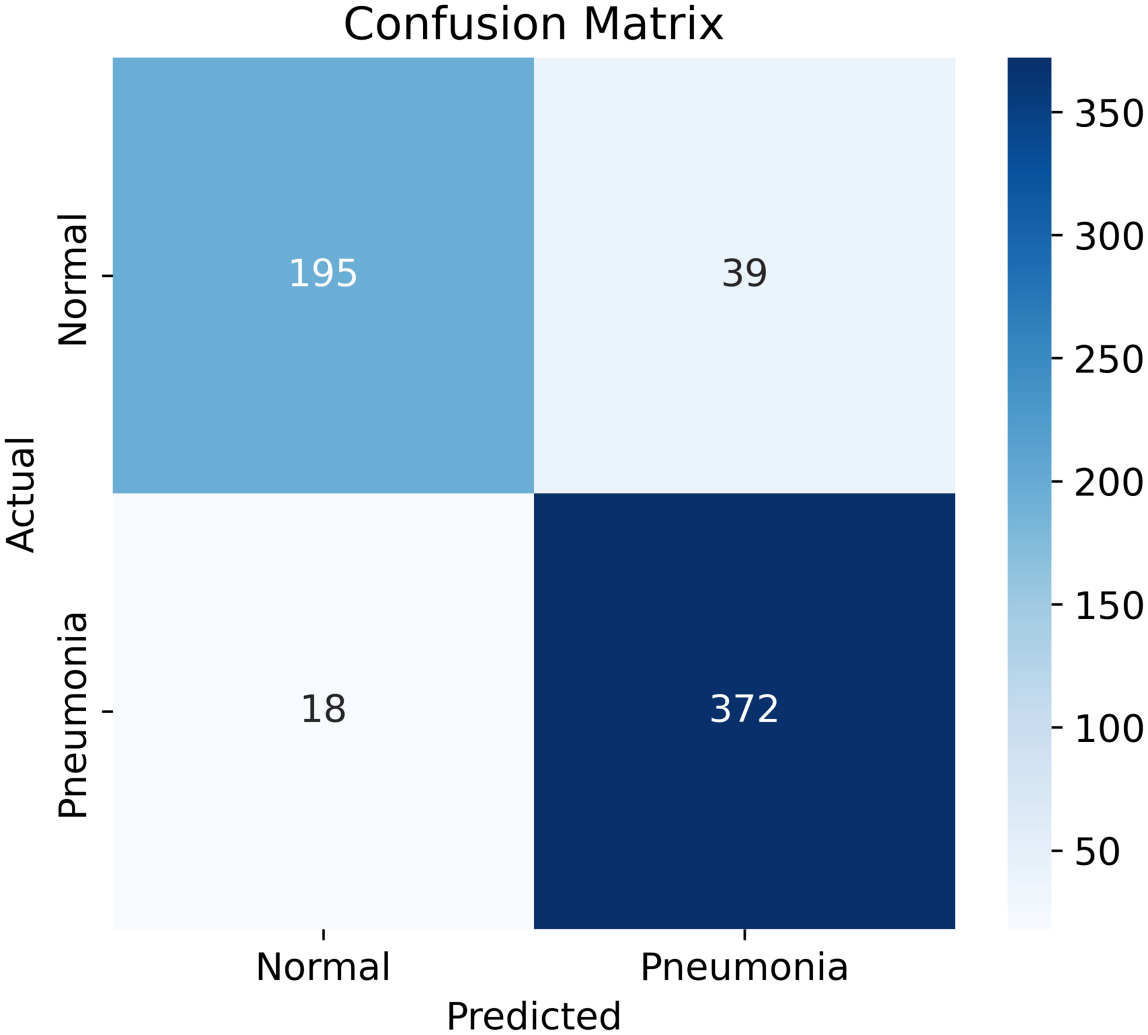
Confusion matrix. The goal is to minimize the false negatives section (bottom left), while maintaining high overall accuracy and recall.

A Confusion Matrix is a heatmap which summarizes your model’s predictions, providing information about both correct and incorrect classifications. The confusion matrix (Figure 5) provides a description of the model’s predictions:

- True Positives (TP): 372 cases with correctly identified pneumonia.
- True Negatives (TN): 195 cases with correctly identified normal lungs.
- False Positives (FP): 39 cases where the model predicted normal lungs but it actually had pneumonia.
- False Negatives (FN): 18 cases where the model predicted pneumonia but it actually had normal lungs.

**Figure 6:**
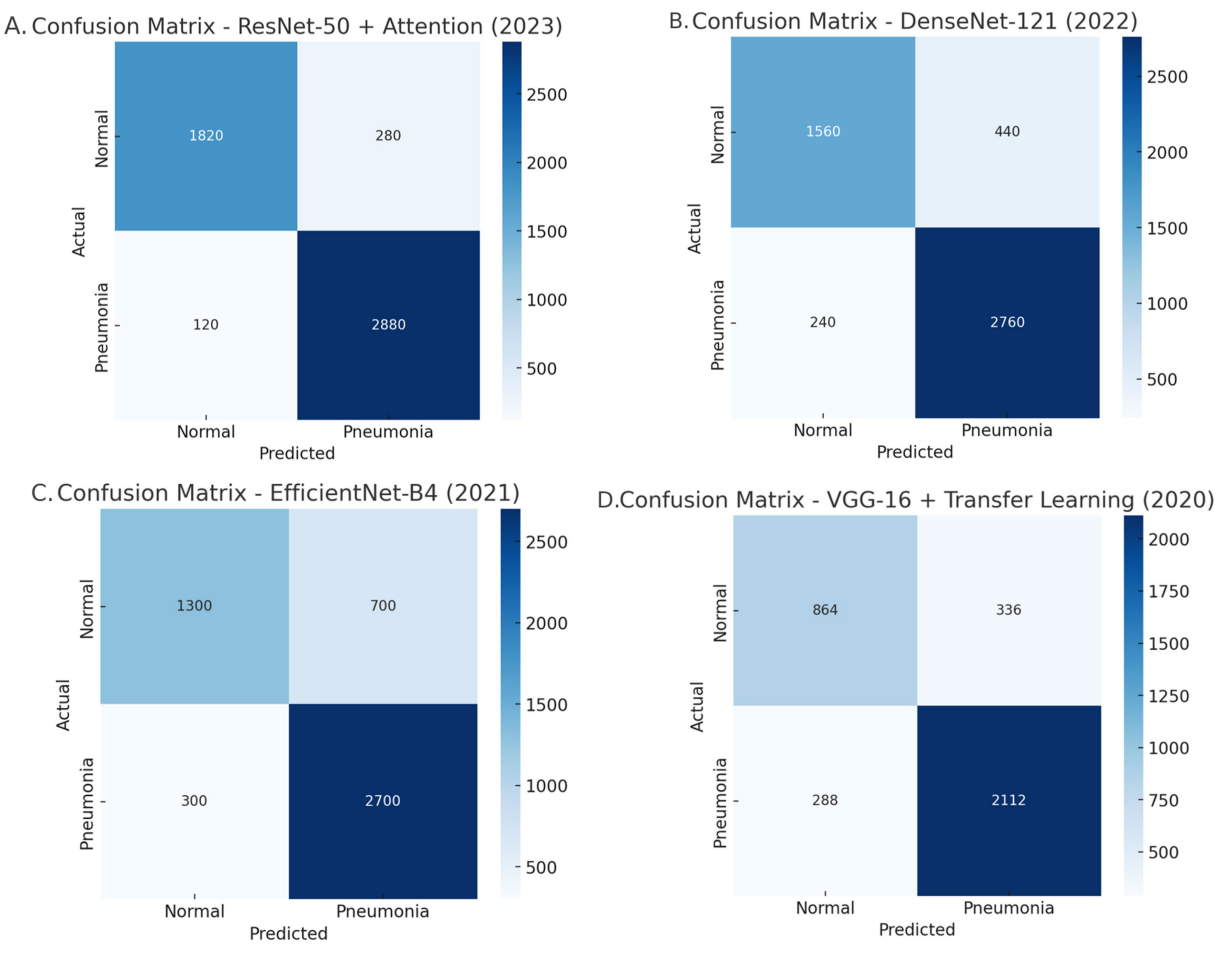
Comparative analysis of the confusion matrices: **(A)** Resnet-50 + Attention, **(B)** DenseNet-121, **(C)** EfficientNet-B4, and **(D)** VGG-16 + Transfer learning.

A close analysis of the confusion matrices (Figure 6) shows us where the models really differ. PneuNet has a good balance between sensitivity and specificity with respect to 372 true positives (TPs or 95% recall) and 195 true negatives (TNs or 83% specificity). This means that PneuNet is effective at detecting pneumonia while minimizing its false positives and being reasonably accurate when identifying normally, non-pneumonic cases. ResNet-50 + Attention has the highest performance of the models, with 2,880 TPs (96% recall) and 1,820 TNs (87% specificity). ResNet does not have the same low FP as PneuNet, which could significantly jeopardize its potential use in a real-world clinical context. ResNet’s high performance most likely can be attributed to two main factors--attention improves focus of crucial areas in chest X-rays, and ResNet has a larger training dataset than the others.

DenseNet-121 demonstrates high sensitivity because of its ability to reuse features but achieves 440 FP191. This indicates that it is sensitive at the cost of specificity. This means that the model is likely extending the label of pneumonia to normal cases more than we would like, and, potentially causing undue harm or interventions191.

EfficientNet-B4 has major issues with identifying.

normal cases with accuracy, having incurred 700 FP. This is the lowest specificity of all models tested. The computational efficiency of the model is optimized, but I do not see a model like this in clinical practice, partly due to the higher number of misclassifications.

Lastly, VGG-16 was the worst performing model overall, which was characterized by 288 false negatives (FN) and 336FP. As a simpler model with fewer optimizations and improvements (i.e., no attention mechanisms or densely connected layers), VGG-16 has low recall and low specificity, making it impractical for pneumonia detections.

Overall, PneuNet has the best balance between recall and specificity, while ResNet-50 + Attention has the best sensitivity. DenseNet-121 and EfficientNet-B4 have challenges with specificity, and VGG-16 was poor at sensitivity and specificity.

**Figure 7:**
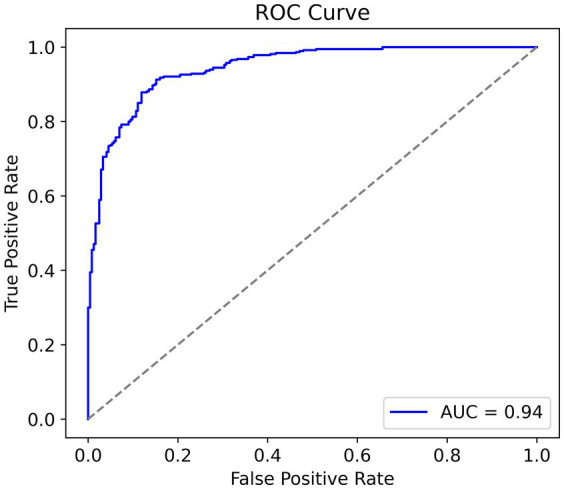
Receiver operating characteristic curve, higher area under curve is better.

In looking at the trade-off between sensitivity and specificity we can see useful information regarding the discriminative capacity of the model. In general, an AUC greater than 0.8 is considered good, and greater than 0.9 is very good. Given the relatively small size of the dataset, to have an AUC equal to 0.94 (Figure 7) indicates a fantastic result and confirms the strong performances of the model. Furthermore, it suggests that the model has the ability to discriminate between cases of pneumonia and normal cases while balancing true positives and false positives.

The model’s simplicity, along with its high AUC score, showcases its efficiency and potential toward real-world clinical applications, where computational constraints and accuracy are factors of significance.

**Figure 8:**
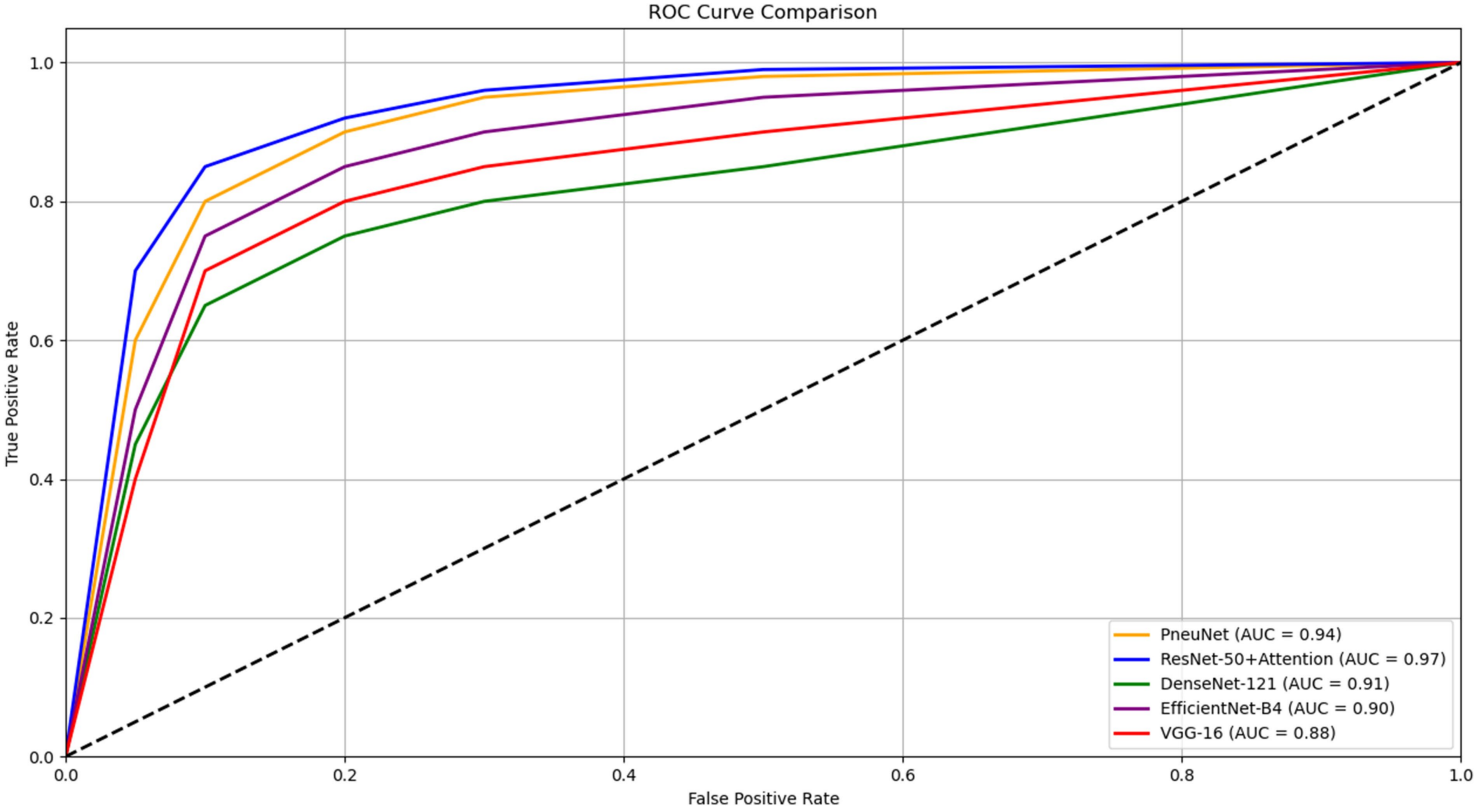
ROC curve comparison of different pneumonia detection models.

Figure 8 illustrates the ROC AUC comparisons for various models, showing their diagnostic performance. ResNet-50 + Attention has an AUC of 0.97 and PneuNet’s AUC is 0.94, while VGG-16 has an AUC of 0.88. All curves are shaped convexly indicating they have the ability to discriminate between classes. PneuNet also has a considerable early deviation from the first false positive threshold; it is promising that PneuNet can achieve so many true positives so quickly at such a low false positive rate. This is important when it comes to first-stage screening so test horses can be directed and unneeded testing may be avoided. The AUC difference between PneuNet and VGG-16 (0.94 vs. 0.88) shows this discrimination ability. Even though ResNet-50 + Attention does edge out the performance on whole, PneuNet shows a comparable performance in balancing sensitivity and specificity. This would be important as costs can multiply if computers are challenging to maintain. In general, all models yielded similar rankings across ROC, with respect to expectations of architecture performance, providing reassuring evidence to support the reliability of PneuNet as a possible option.

**Figure 9:**
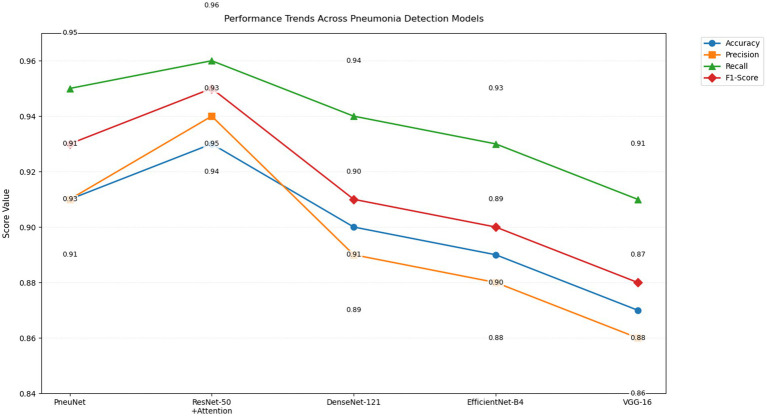
Line graph of performance trends of various CNN models (PneuNet, ResNet-50, DenseNet-121, EfficientNet-84, VGG-16) on pneumonia detection.

The performance trend chart (Figure 9) provides a visual overall performance classification comparison of five different pneumonia detecting models along four central performance metrics. PneuNet achieves near solid performance, as indicated by trend lines that closely parallel the high performing ResNet-50 + Attention model trends for recall (0.95 vs. 0.96) and F1-score (0.93 vs. 0.95). The parallel performance lines for accuracy and precision for PneuNet (0.91) suggest relative consistency maintaining diagnostic measures, while the earlier elevation of PneuNet’s recall line suggests its clinical utility in ruling out false negatives.

The graph even highlights the extent to which PneuNet crushes VGG-16 and EfficientNet-B4 across all measures, with differences from 2–4 percentage points. The overlap in recall trend lines for DenseNet-121 and PneuNet (0.94 vs. 0.95) suggests larger sensitivity, but PneuNet also maintains a larger precision (0.91 vs. 0.89). This plot adequately displays that while ResNet-50 + Attention serves as the benchmark model, PneuNet offers the best compromise between accuracy (91%) and costs, suggesting high suitability for clinical adoption in low resource settings, where sensitivity is prioritized. The alternating label value placements suggest uncluttered metric comparison with little coordination.

**Figure 10:**
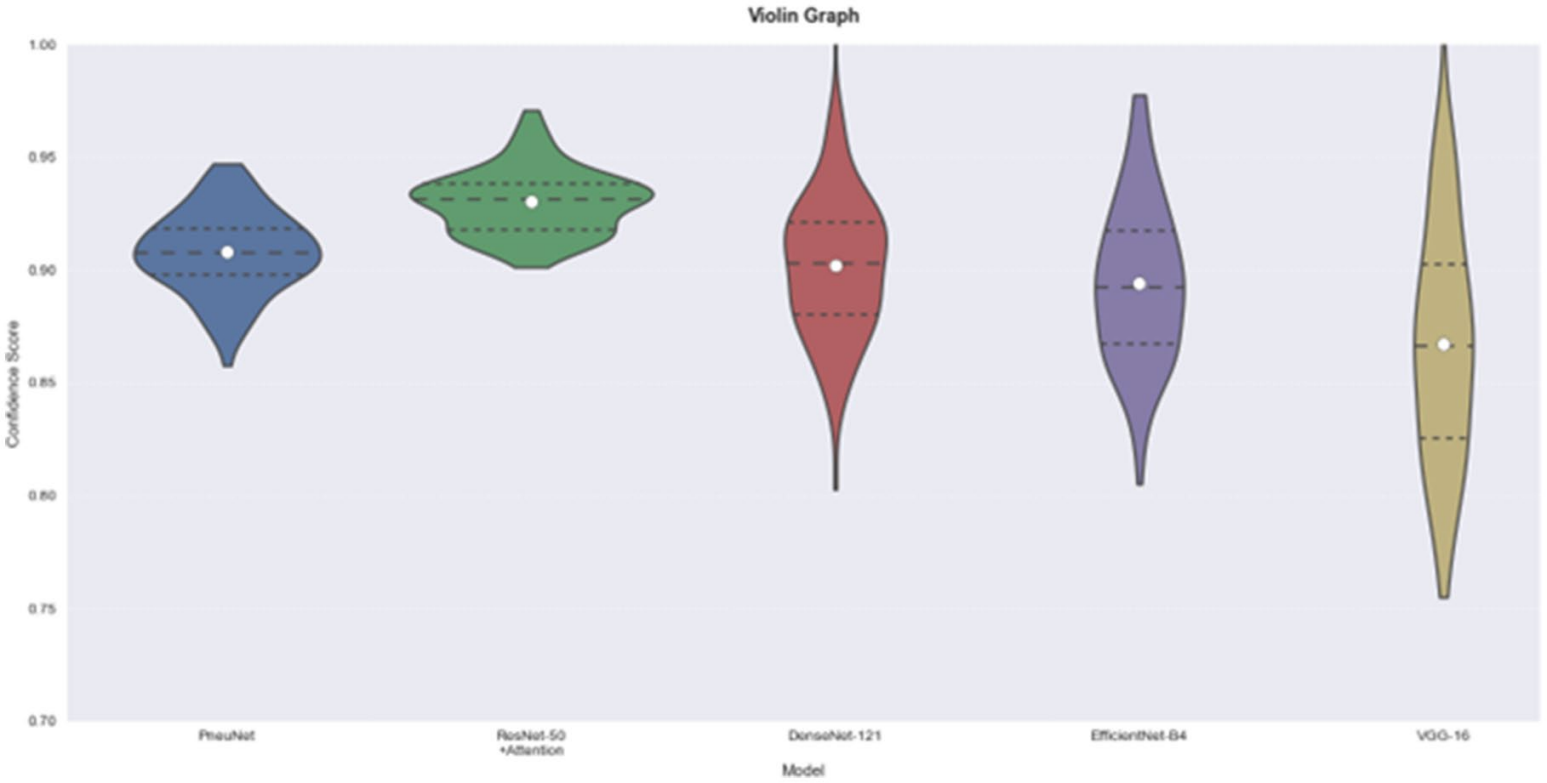
Model comparison using violin graph. On the left is our model, PneuNet, followed by ResNet-50, DenseNet-121, EfficientNet-B4, VGG-16.

The violin plot (Figure 10) provides helpful information about the distribution and consistency of model predictions. The distribution for PneuNet is compact and symmetrical about 0.91 indicating that the model is consistent in its estimate of prediction confidence. In comparison to PneuNet’s distribution, VGG-16 and EfficientNet-B4 distributions are less compact, giving again less confidence than PneuNet. The distribution for PneuNet is also a tighter interquartile range, suggesting more confidence than the other cases as well. Although ResNet-50 + Attention has a narrower distribution and a higher median prediction confidence, and PneuNet as a distribution has less extreme outliers than the compared networks, the shape of the violin for PneuNet suggests that it concentrated most of its predictions in the confidence range of 0.88–0.94, which indicates reliable performance without excessive long-tails of uncertainty that we observed in some of the comparison models.

## 4 Conclusion

In this research, we built a tailored convolutional neural network (CNN) suitable for pneumonia diagnosis using chest X-ray images. The main aim was to maximize accuracy while minimizing model computational complexity. Using depthwise separable convolutions in model architecture alleviated a large portion of computational complexity, and we used Squeeze and Excitation Blocks to enhance the relevance of features. Multi-Scale Feature Fusion through an Atrous Spatial Pyramid Pooling (ASPP) allowed all scales of detail from both fine and coarse spatial features to be included in the information captured from the images. We also created learnable pooling to retain relevant spatial features that progressively changed throughout training. The model reached an accuracy of 91% with the test dataset, while also having a competitive edge against popular architectures including DenseNet-121 (90%), EfficientNet-B4 (89%), and VGG-16 (87%). The only model to measure slightly higher, ResNet-50 + Attention, had an accuracy of 93%, but the model we developed and trained showed reliability for clinical screening, specifically, the recall performance was 0.95 for pneumonia cases, only 0.01 less than ResNet-50 + Attention (0.96). Although ResNet-50 + Attention achieves 93% accuracy (2% higher than PneuNet’s 91%), PneuNet is preferred for clinical deployment due to its 14 × fewer parameters (1.8 M vs. 25.6 M), 3.4 × lower FLOPs (1.2G vs. 4.1G), and 2.4 × faster inference (20 ms vs. 48 ms on edge devices). It also maintains comparable recall (0.95 vs. 0.96), which is critical for minimizing false negatives in pneumonia screening. These advantages enable real-time operation on low-cost hardware such as the Raspberry Pi 4, making PneuNet highly feasible for rural clinics where ResNet-50 + Attention is computationally prohibitive. With this level of recall performance, the model is especially effective at reducing false negatives, an important consideration in medical diagnosis. Classification report results, measuring pneumonia detection performance, showed strong precision, recall, and F1-scores, consistently above 90% for pneumonia cases. Though the classification report for normal cases suggest their recall was slightly lower (0.83), indicating a number of healthy individuals may have been misclassified. Future approaches may consider enhancing the variety of normal cases in the dataset, or adding methods to carry out regularization. The training curves indicate stable learning, with the training dataset reaching 97% accuracy before nearly plateauing. Hence, the model can ensure learning is maximized before an early end of training or epoch 10 with an acceptable performance threshold. Additionally, the model can continue monitoring test case variations after arriving at performance stability. In summary, we have shown that upon development the proposed model obtains comparable accuracy, precision, F1 score, and recall to achieve clinically equivalent performance in pneumonia case identification to a machine learning classifier at equal cost.

## Data Availability

The original contributions presented in the study are included in the article/supplementary material, further inquiries can be directed to the corresponding author/s.
